# Mr. Mantal, on Gout

**Published:** 1804-12-01

**Authors:** James Mantal

**Affiliations:** Bromley High Elms


### To the Editors of the Medical and Physical Journal.

GENTLEMEN,

THE observations which have occurred to me on perusing Dr. Kinglake's Dissertation on Gout, are by no means inimical to the mode of cure there recommended; for, I think, that with a due degree of caution, it is the true remedy indicated by the nature of the disease; and which will finally prevail over the prejudices that have in all ages obstructed the free use of it; though it seems that in a few instances, which ought to have weight from the celebrity of their names in medical science, (I allude principally to the examples of Dr. Harvey and Dr. Gregory) it has been adopted without fear, and been attended with no bad effects, that could in fair reason be attributed to it; yet they have not been sufficient to open the eyes of the generality of medical professors, or to induce an imitation of the practice. Dr. K. is, I think, much to be commended for having introduced it to the notice and consideration of the medical world; and I hope his theory of the disease, as well as the mode of cure he recommends, will undergo a thorough investigation. I have no wish to raise frivolous objections either to one or the other; but with respect to his theory, it seems to me a mere gratuitous assumption, that gout is a disease of the ligaments and tendons only, as no proof is offered to establish so necessary a position; necessary, I mean, to his theory; because on the truth of this fact the impossibility of constitutional or visceral gout is made to depend. I might, I think, as plausibly maintain, on the other side of the question, that gout cannot be a disease of the ligaments and tendons, because there is no connection or association between the ligamentous and tendinous structure, and that of the visceral organs, its primary seat. But I apprehend such a mode of arguing will have little weight with Dr. Kinglake, or any philosophic mind, towards establishing what, nevertheless, I believe to be true, namely, that gout is not a disease of the ligaments and tendons.

I rather agree with those physicians, who consider it as a disease of the membranes that cover the joints, which from their situation, their perpetual extension and contraction on the frequent motion of the joints, are liable to greater mobility, and consequently more susceptible of inflaminflammation than the membranes in their vicinity; and from the similarity of their structure with the membranous parts of the stomach and liver, may have their action associated with those important vital organs. Perhaps Dr. Kinglake would find it difficult to assign a reason, why, when gouty inflammation attacks the tendons and ligaments of the great toe, and of the ancle and knees in succession, or at the same time, the intermediate parts of that structure should escape without injury. But there seems no room for such an objection on the view of the disease which I have presented. Now supposing, (and I am inclined to think, that gout is produced on the extremities in this manner, though I am ready to own that the subject is very obscure, and perhaps will never be explained) supposing, I say, that some important viscus, or some membranous parts of it, become affected with a degree of torpor in consequence of cold, owing perhaps to a deficient secretion, caused by a want of due stimulation. the usual stimuli having lost a part of their effect, or the affected viscus a part of its irritability, what is the consequence to be expected? By a law of the animal economy, as it is well explained in Zoonomia, (a work which I may observe, by the way, has not yet received due attention, nor its author his due honours,) the torpor, after a certain time, should be followed by inflammation, where the vital powers are sufficent for that purpose; and by another law of the system, that inflammation does not take place on the part affected with the torpor, but is immediately transferred to a less important part, that is, to the extremities, as far as the sympathetic action can be carried, and as far as possible from the seat of vital motion, which is always the last to suffer, and does not in this disorder materially suffer, so long as it retains the power of making that salutary effort, and of relieving the more important viscera, by a metastasis of the inflammation. When gout attacks the stomach, I believe it is always the torpor which the vital powers are insufficient to remove; and which sometimes, by association or sympathy of contiguity, extends to the heart, and puts a sudden period to life. In this stage of the disorder, opium and the hot spices, by enlivening and giving a temporary force to the oppressed vital powers, produce not only a cessation of the torpor, but an increased activity, in proportion to the previous degree of it; which the vires medicatrices naturæ, exerting themselves, remove to the extremities; and it there constitutes a

 *salutary*

*salutary* disease for the moment, or one of very little consequence, compared with that which it succeeded.

With respect to the *practice* of Dr. Kinglake, my only difference with him is, that I think some caution is necessary in the application of his remedy.

When the inflammation is completely formed on the extremities, (not at the first feeling a painful stiffness of the affected joint) and when the visceral uneasiness, arising from their torpor, which I believe always precedes the inflammation of the extremities, has entirely subsided, I see no reason why the remedy recommended by Dr. Kinglake should not be immediately had recourse to, and, as I am convinced, without any danger of repulsion, because then the sympathy, I apprehend, ceases, and the associated action between the affected viscera and the extremities is as much dissevered, as if no such sympathy had ever existed; the viscera having recovered their healthy state, (*generally*, I believe always, except where the constitution is broken down by the violence and duration of former attacks,) as soon as the inflammation is fixed on the extremities. Therefore we may surely proceed immediately to the cure of it; and that we may do so without danger to life, is abundantly evinced by that cloud of witnesses, brought forward at the end of the volume; for which we are indebted to the science of Dr. Kinglake, and the successful boldness of his numerous correspondents. From whose testimony it appears, that in all the variety of ages and constitutions, and in habits which had experienced the tyranny of this disorder without controul for a great number of years, the relief obtained by the simple application of a cold fluid, was immediate, safe, durable, and perfect. Dr. Kinglake (by the bye I think, rashly) has challenged the whole world to produce an instance of failure, or of a calamitous issue; and not one has been produced, or produced only to be refuted.

The case of Mr. Baker, of Uxbridge,* ought by all means to be laid before the public. It would be the greatest sacrifice to false delicacy, not to say great inhumanity, to refuse a compliance with the request which has been made for that purpose, when so much instruction is probably to be obtained from it, if it be related without prejudice, and with a strict attention to the minutest particulars. I believe

---

* We perfectly agree with Mr. M. and believe the public will not be disappointed. Ed.

believe it will be found to confirm the necessity of the caution, which I have insisted on, that the remedy should not be applied till the inflammation is completely formed on the joints, when the sympathy or associated action is supposed no longer to exist. If ice water be applied before that period, the torpor from so great a degree of cold would not only instantly put a stop to what may be called the salutary inflammation of the extremities, which is just beginning to form, but would perhaps be immediately communicated to the viscus, the original seat of the disorder, the associated action between them not being entirely dissevered.*

I have heard of a case somewhat similar; the particulars of which might, perhaps, yet be redeemed from oblivion. It is that of the Rev. Mr. Smith, who was Rector of Grinstead, in Colchester, nearly twenty years ago, who, I believe, sprained his ancle, and treated it as a sprain with cold applications. But it was supposed to have brought on sympathetic gout in the stomach, and was certainly attended with fatal consequences. If inflammation was the consequence of the sprain, and, in spite of that, the gouty torpor seized the vital parts, it might receive some explanation from that law of animated Nature, called *reverse sympathy*, which more frequently occurs in weak than in robust constitutions, as well explained and illustrated in various parts of Zoonomia. If Mr. Newell, who, I believe, did at that period (as he has done ever since) administer the comforts of health, under Divine Providence, to the inhabitants of Colchester and its vicinity, should deem it a case of any importance towards throwing light on an interesting and momentous question, which must be considered at present as undecided, and will have the goodness to collect and communicate the particulars of it to the public, I will venture to affirm, that your numerous readers will be no less gratified with an impartial and minute statement of an interesting case, than instructed by the justness and philosophical correctness of his remarks, if he should think proper to add any to the statement. I am, &c.

*Bromley High Elms, Oct.* 15, 1804. JAMES MANTAL.

---

* A Case has been sent to us where these cautions were not attended to, and the termination was fatal. As our Correspondent requests Dr. Kinglake's opinion on his manner of treating his patient, we shall send Dr. K. a copy of the Case, and expect his answer. Ed.

